# AjTEAD1 Targets AjCyclin E to Promote Cell Proliferation During Intestinal Regeneration in *Apostichopus japonicus*

**DOI:** 10.3390/biom16050642

**Published:** 2026-04-25

**Authors:** Chuili Zeng, Xu Zhan, Ke Xiao, Chenghua Li

**Affiliations:** 1 State Key Laboratory of Agricultural Products Safety, Ningbo University, 818 Fenghua Road, Ningbo 315211, China; zengchuili@nbu.edu.cn (C.Z.); z15757862257@gmail.com (X.Z.); 2301130073@nbu.edu.com (K.X.); 2 Laboratory for Marine Fisheries Science and Food Production Processes, Qingdao National Laboratory for Marine Science and Technology, Qingdao 266071, China

**Keywords:** *Apostichopus japonicus*, intestine regeneration, cell proliferation, *TEAD*, *Cyclin E*

## Abstract

TEA domain transcription factors are critical regulators of tissue development and regeneration in mammals, yet their roles in aquatic invertebrate regeneration remain poorly understood. Here, a full-length cDNA encoding a putative transcriptional enhanced associate domain protein 1 (TEAD1) ortholog in *Apostichopus japonicus* (*AjTEAD1*) was cloned and characterized. The open reading frame (ORF) of *AjTEAD1* is 1344 bp, encoding a polypeptide of 447 amino acids with a conserved TEA domain (Asp^40^–Leu^111^) and a protein-binding domain (Gly^231^–Asp^446^). Function analysis demonstrates that AjTEAD1 is essential for intestinal regeneration. AjTEAD1 expression was significantly upregulated during the regeneration process. Functional impairment of AjTEAD1 suppressed intestinal regeneration and attenuated cell proliferation. At the molecular level, we identified the cell cycle gene in *A. japonicus* (*AjCyclin E*), whose expression pattern coincided with that of *AjTEAD1* and was downregulated following *AjTEAD1* knockdown. Dual-luciferase reporter assays further confirmed that AjTEAD1 binds to specific sites in the *AjCyclin E* promoter and transcriptionally activates its expression. In summary, our study reveals that AjTEAD1 promotes cell proliferation and drives intestinal regeneration in *A. japonicus* by directly upregulating *AjCyclin E* transcription. These findings identify the TEAD–Cyclin E axis as a key regulator of echinoderm regeneration, shedding new light on the regenerative processes and cytological mechanisms in economically important species.

## 1. Introduction

Regeneration is a widespread developmental phenomenon that serves as a vital adaptive strategy, enabling organisms to recover from injury and respond to environmental challenges. It encompasses a spectrum of processes, ranging from basic wound healing to the reconstruction of complex organs. Regenerative capacity varies markedly across animal phyla: invertebrates generally exhibit strong restorative potential, whereas vertebrates are often restricted to regenerating specific tissues or organs [1]. In recent years, the field of regeneration research has extended beyond classical model organisms to include a diversity of non-traditional systems. This comparative approach seeks to uncover conserved principles of regeneration by examining mechanisms across evolutionarily distant taxa. Among these, echinoderms occupy a key phylogenetic position as deuterostomes closely related to chordates, offering valuable insights into the evolution and developmental biology of regeneration [2]. In particular, sea cucumbers (Holothuroidea) have emerged as an exceptionally attractive model due to their ability to rapidly and completely regenerate complex organs like the digestive system following evisceration [3]. This system provides a unique research model for studying injury-induced cellular plasticity, tissue reconstruction, and regenerative regulatory mechanisms. Such research not only deepens our understanding of regenerative mechanisms in deuterostomes but also offers potential insights for studies in regenerative medicine.

The sea cucumber (*Apostichopus japonicus*) is an economically important aquaculture species in Asia, possessing remarkable regenerative capabilities. Under stress conditions such as pollution, high temperatures, or overcrowding, it can undergo evisceration (including the intestines, respiratory tree, and gonads), and fully regenerate these structures once environmental conditions improve [4]. This highly repeatable process can be artificially induced, providing a valuable model for studying regeneration. Research on intestinal regeneration in sea cucumbers spans over nine decades, with histological and cellular insights revealing roles for proliferation, autophagy, apoptosis, and dedifferentiation [5]. Among these processes, cell proliferation serves as the key mechanism for maintaining tissue homeostasis during intestinal primordium formation in sea cucumbers. It drives the expansion of the mesentery terminus by supplying new cells. However, systematic studies on its regulation during intestinal regeneration remain preliminary, and the underlying regulatory network has yet to be fully elucidated. Although existing research, such as on the Wnt pathway and the AjFGF4/AjFGFR2–ERK–cell cycle axis [5,6], has begun to outline parts of the regulatory framework, a comprehensive understanding is still lacking. Therefore, further investigation into the mechanisms controlling cell proliferation in sea cucumber intestinal regeneration will not only advance fundamental knowledge in regenerative biology but also provide broader insights into tissue repair and regeneration.

During the process of cell proliferation involved in regeneration, the transcriptional regulatory network acts as a master regulator [7]. The transcriptional enhanced associate domain protein (TEAD), as highly conserved downstream effectors of the Hippo signaling pathway, have emerged as pivotal molecules linking the cellular microenvironment to tissue regeneration [8]. This is due to their ability to integrate intracellular and extracellular signals, such as mechanical forces and cell contact, and directly regulate the expression of genes involved in the cell cycle, apoptosis, and cell fate determination. In various tissue regeneration models, TEAD has been proven to function as a critical molecular switch that coordinates the fundamental paradox between “proliferative expansion” and “functional differentiation”. For instance, during pancreatic β-cell regeneration, TEAD1 not only drives the expression of identity-related genes (e.g., *Pdx1*) in mature β-cells to maintain function, but also suppresses the cell cycle by regulating genes such as *Cdkn2a*, thereby ensuring that regenerated cells acquire full physiological functionality rather than undergoing uncontrolled proliferation [9]. Similarly, in the development and regeneration of peripheral nerve myelin, TEAD1 is essential for driving the functional differentiation of Schwann cells, such as cholesterol synthesis for myelin formation [10]. Collectively, these studies reveal that TEAD does not act merely as an on-off switch for proliferation, but rather as a precise “balancer” that ensures regeneration occurs at the right time, in the right place, at the appropriate scale, and with the correct cell type. However, current understanding of TEAD function is largely derived from studies in vertebrates with limited regenerative capacity or under homeostatic renewal. In invertebrates with remarkable regenerative abilities, such as sea cucumbers, whether and how TEAD regulates de novo reconstruction of entire complex organs remains an unexplored area, including its mechanisms and regulatory networks.

Our preliminary qPCR analysis revealed that *AjTEAD* expression dynamics during key phases of intestinal regeneration in sea cucumbers correlate closely with fluctuations in cell proliferation, suggesting a potential regulatory role for *AjTEAD* in this process. Building on these observations, we hypothesized that *AjTEAD* drives and coordinates intestinal regeneration by responding to injury signals and modulating downstream gene networks involved in cell cycle and proliferation. In this study, we demonstrate that *AjTEAD1* is significantly upregulated during intestinal regeneration in *A. japonicus* and promotes regeneration by enhancing cell proliferation. Mechanistically, AjTEAD1 binds to the promoter of the cell-cycle gene *AjCyclin E* and activates its transcription. Interference with AjTEAD1 suppressed both Cyclin E expression and cell proliferation, thereby impairing intestinal regeneration. Collectively, these results establish that AjTEAD1 promotes intestinal regeneration through transcriptional activation of AjCyclin E, revealing a conserved TEAD-Cyclin regulatory axis in echinoderms and providing new insights into regeneration mechanisms.

## 2. Materials and Methods

### 2.1. Ethics Statement

The BALB/c mouse and *A. japonicus* utilized in this study were commercially bred, and all experiments followed the National Institutes of Health’s Guide for the Care and Use of Laboratory Animals. The study protocol was authorized by Ningbo University’s Experimental Animal Ethics Committee (No. NBU.ES-2021-11180) with an approval date of 15 March 2021.

### 2.2. Animals and Treatment

Healthy adult specimens of *A. japonicus* (weighing 100 ± 5 g) were sourced from Dalian Pacific Aquaculture Company. Prior to the experiment, they were kept in natural seawater (salinity 28 ± 1, temperature 16 ± 1 °C) and received a daily ration of commercial feed for one week. After this acclimatization period, evisceration was triggered by administering 2–3 mL of 0.35 M KCl into the coelomic cavity. Subsequently, the animals were transferred to seawater tanks to facilitate intestinal regeneration. Control groups consisted of uneviscerated individuals kept under identical conditions.

### 2.3. Detection of EdU-Positive Cells by Immunohistochemistry and Flow Cytometry

To examine cell proliferation dynamics and tissue distribution during intestinal regeneration, 5-Ethynyl-20-deoxyuridine (EdU) labeling coupled with immunohistochemistry and flow cytometry were employed. Intraperitoneal injections of EdU were performed 24 h prior to each sampling time point. For immunohistochemical analysis, a dorsal incision exposed the sea cucumber’s coelomic cavity, enabling mesentery separation from the body wall and intestine. In non-eviscerated individuals, the mesentery was carefully removed with fine scissors. The complete mesentery, extending from the anterior (esophageal) to the posterior (cloacal) end, was harvested, immersed in frozen section medium, and preserved at −80 °C. Subsequently, 6 μm thick cryosections were cut on a Leica CM1900 cryostat at −20 °C. The sections were then fixed with pre-cooled acetone (4 °C) for 15 min, rinsed for 10 min in PBST (0.5% Tween 20 in PBS), and permeabilized via a 30 min treatment with 0.5% Triton X-100 (Cat#648462, Merck KGaA, Darmstadt, Germany). After additional washing, these slices were reacted with EdU click reaction buffer for 30 min at 37 °C in darkness. Subsequently, nuclei staining was performed with Hoechst 33342 (Cat#C1025, Beyotime, Shanghai, China) for 10 min, followed by visualization using a ZEISS Axio Observer 7 (Carl Zeiss Microscopy GmbH, Jena, Germany). Regenerative area dimensions were measured with ImageJ software (v1.54r, National Institutes of Health, Bethesda, MD, USA). A minimum of three non-adjacent sections per animal were analyzed to determine average rudiment size. For flow cytometric assessment, single cells were harvested from regenerating tissues collected at various time points via digestion with 0.05% trypsin (Cat#T4799, Sigma-Aldrich, Darmstadt, Germany). The samples were pelleted by centrifugation at 800× *g* for 5 min and subsequently fixed with 4% formaldehyde (Cat#100496, Sigma-Aldrich, Darmstadt, Germany) for 10 min, washed three times with PBST, and treated with EdU click reaction mixture reaction reagent (Cat#C0071S, Beyotime Biotechnology, Shanghai, China) for half hour in the dark. Following a final wash, proliferative activity in 1 × 10^5^ cells was quantified using an Agilent NovoCyte flow cytometer (Agilent Technologies, Inc., Santa Clara, CA, USA). Data analysis was performed via FlowJo software (v10.6.1).

### 2.4. Recombinant Protein Expression and Antibody Production

For recombinant production of the *A. japonicus* TEAD1 (AjTEAD1) protein, the pET32a(+)-*AjTEAD1* vector was assembled and introduced into *E. coli* Rosetta (DE3) host cells. Initially, pET32a(+) underwent digestion with EcoRI and XhoI. The *AjTEAD1* open reading frame (1344 bp) was PCR-amplified from *A. japonicus* cDNA with primers detailed in Table 1. Following purification, the amplicon was inserted into the restricted vector by homologous recombination using a seamless cloning system (Cat#CT101-01, TransGen Biotech, Beijing, China), yielding the recombinant construct in *E. coli* DH5α. DNA sequencing (Sangon Biotech, Shanghai, China) verified the construct. Upon confirmation, transformation of the construct into *E. coli* Rosetta (DE3) was performed, followed by overnight induction at 18 °C with IPTG. Purification of the expressed rAjTEAD1 was achieved using Ni-NTA Agarose (Cat#30230, QIAGEN, Hilden, Germany), with subsequent analysis by SDS-PAGE. As a prerequisite for antibody preparation, the purified protein was subjected to enterokinase digestion (Cat#E8352, Solarbio, Beijing, China). For immunization, six BALB/c mice received four immunizations with the processed rAjTEAD1. The first immunization consisted of an intraperitoneal injection of 200 μL of an emulsified preparation containing 50 μg of protein combined with an equal volume of Complete Freund’s Adjuvant (Cat#HY-9007-81-2, MedChemExpress, Monmouth Junction, NJ, USA). Two weeks later, a secondary immunization was administered using the same protein amount emulsified with Incomplete Freund’s Adjuvant (Cat#P2031, Beyotime Biotechnology, Shanghai, China). For subsequent boosts, 0.2 mL of adjuvant-free protein solution was injected into the tail vein. At 7 days post-final immunization, blood was obtained via retro-orbital bleeding. The specimens were first kept at room temperature for 2 h and subsequently incubated at 4 °C overnight. Following centrifugation at 10,000 rpm for 5 min, the serum fraction was collected. Mouse IgG was then purified from this serum using a protein G-agarose column (Cat#HY-K0214, MedChemExpress, Monmouth Junction, NJ, USA), with characterization performed by ELISA and Western blotting.

### 2.5. Protein Extraction, SDS-Page and Western Blot

Protein extraction from *A*. *japonicus* mesentery and regenerated intestinal tissues in each group was performed by homogenization at −10 °C with a tissue disruptor. Subsequent lysis was carried out using cell lysis solution (Cat#P0013, Beyotime, Shanghai, China). Protein concentrations were quantified with a BCA Protein Assay Kit (Cat#CW0014S, CWBIO, Taizhou, Jiangsu, China). For each sample, 50 µg of protein was resolved on 12% SDS-polyacrylamide gels in a Tris-Glycine buffer system, with electrophoresis conducted at a constant voltage of 120 V. Following electrophoresis, proteins were transferred to nitrocellulose membranes (Cat#HATF04700, MedChemExpress, Monmouth Junction, NJ, USA) via wet transfer in a mini-transfer tank (Bio-Rad Laboratories) using NcmBlot Rapid Transfer Buffer (Cat#WB4600, NCM Biotech, Suzhou, China). After transfer, membranes were placed in blocking solution (5% non-fat milk in TBST) for 2 h at ambient temperature. Primary antibody incubations (antibodies listed in Table 2) were conducted overnight at 4 °C. Following extensive washing with TBST (three times), the membranes were incubated for 2 h at room temperature with HRP-tagged secondary antibodies (Table 2) and subsequently washed another three times. Immunocomplexes were visualized via enhanced chemiluminescence using NcmECL Ultra (Cat#P10200, NCM Biotech, Suzhou, Jiangsu, China) and documented with an Aplegen Omega Lum C imaging system (Gel Company, San Francisco, CA, USA). Signal intensities were quantified densitometrically using ImageJ software (v1.54r, National Institutes of Health, Bethesda, MD, USA). Mean values from three independent experiments were determined, and target protein expression was normalized to tubulin levels.

### 2.6. RT-qPCR (Real-Time Quantitative Polymerase Chain Reaction) Analysis

Total RNA was isolated from 100 mg of intestinal regeneration tissues at normal status and various regeneration stages (2, 7, 12 and 17 days post evisceration, dpe) using Trizol reagent (Cat#T9108, Takara Bio Inc., Kusatsu, Shiga, Japan) following the manufacturer’s instructions. RNA concentration was quantified on a NanoDrop 2000 spectrophotometer (Thermo Scientific, Waltham, MA, USA), and its integrity verified via 1.5% agarose gel electrophoresis. Genomic DNA (gDNA) was removed by treating 1 µg of total RNA with 1 unit of DNase I for 15 min at 37 °C. Complementary DNA (cDNA) synthesis was performed using the PrimeScript™ RT Reagent Kit (Cat#RR047A, Takara Bio Inc., Kusatsu, Shiga, Japan). RT-qPCR analysis was conducted on an Applied Biosystems 7500 instrument employing TB Green Premix Ex Taq™ II (Cat#CN830A, Takara Bio Inc., Kusatsu, Shiga, Japan) with 10-fold serially diluted cDNA. Oligonucleotide primers targeting *AjTEAD1* and *AjCyclin E* were designed using Primer 5 software (Table 1). Reactions (20 µL) contained 10 µL SYBR Green I Master, 2 µL diluted cDNA, 0.4 µL each primer (10 µM), 0.4 µL ROX II, and 6.8 µL RNase-free water. Cycling parameters were: 95 °C for 30 s, then 45 cycles of 95 °C for 5 s and 60 °C for 30 s [11]. Each sample was assayed in triplicate across three independent experiments. Expression levels were normalized to *Ajβ-tubulin* and quantified employing the 2^−△△Ct^ method [12].

### 2.7. RNA Interference (RNAi) and Inhibitor Assays

The siRNA oligonucleotides targeting *AjTEAD1* (sequences provided in Table 1) along with a negative control (NC) siRNA were obtained from GenePharma Company (Suzhou GenePharma Co., Ltd., Suzhou, Jiangsu, China). Both siRNA types were diluted in RNase-free water to 20 µM stock solutions. Each 100 µL inoculum comprised 10 µL Lipo6000 (Cat#C0526, Byotiome, Beijing, China), 10 µL of 20 µM siAjTEAD1 or siNC, and 80 µL PBS. Injections were given via the intraperitoneal route every 48 h during intestinal regeneration. The initial administration was performed at 6 h after evisceration, with the final booster at 10 dpe. Mesentery and intestinal specimens were collected at 2, 7, and 12 dpe and stored at −20 °C for RT-qPCR and Western blotting.

### 2.8. Cell Culture

Human embryonic kidney epithelial cells (HEK 293T; ATCC, CRL-3216) were cultured in Dulbecco’s Modified Eagle Medium (DMEM; Cat#10013102, Corning Incorporated, Corning, NY, USA), supplemented with 10% fetal bovine serum (FBS, Cat#10270-106, Gibco, Grand Island, NY, USA). The cells were maintained at 37 °C in a humidified atmosphere containing 5% CO_2_. All cell culture dishes and plates were obtained from Jet Bio-Filtration Co., Ltd. (Cat#CSP010024, Guangzhou, China) and NEST Biotechnology Co., Ltd. (Cat#702003, Wuxi, Suzhou, China). Regular subculturing was performed to ensure optimal cell viability and confluency for subsequent experiments.

### 2.9. Construction and Transfection of Reporter Gene Vectors

Based on the prediction of potential transcription factor binding sites in the *AjCyclin E* promoter sequence using JASPAR (https://jaspar.elixir.no/, accessed on 26 March 2026), the pGL3-*AjCyclin E* reporter vector and pCMV-Flag 2C-*AjTEAD1* vector were constructed using homologous recombination, as detailed below. Firstly, homologous arms were designed upstream of the Kpn I restriction site and downstream of the Xho I restriction site on the pGL3-Basic vector (Table 1). Using sea cucumber genomic DNA as a template, PCR cloning yielded a truncated sequence of the *Ajcyclin E* promoter with homologous arms. Then, following the instructions provided in the pEASY^®^-Basic Seamless Cloning and Assembly Kit (Cat#CU201, TransGen Biotech Co., Ltd., Beijing, China), the Kpn I and Xho I digested pGL3-Basic plasmid was subjected to homologous recombination with the *AjCyclin E* (P1), *AjCyclin E* (P2), *AjCyclin E* (P3), *AjCyclin E* (P4), and *AjCyclin E* (P5) fragments carrying homologous arms. Finally, the sea cucumber pGL3-*AjCyclin E* (P1), pGL3-*AjCyclin E* (P2), pGL3-*AjCyclin E* (P3), pGL3-*AjCyclin E* (P4), and pGL3-*AjCyclin E* (P5) reporter vectors were obtained through transformation, sequencing, and plasmid extraction.

HEK293T cells were dispensed into 24-well plates (1 × 10^4^ cells/well) and grown for 24 h at 37 °C in a humidified 5% CO_2_ incubator. At approximately 70% confluency, transfection was performed with Lipofectamine 6000 (Cat#C0526, Beyotime, Shanghai, China). The transfection mixtures were prepared as follows: Control group: 200 ng pGL3-Basic empty vector + 300 ng pCMV-Flag 2C empty vector + 20 ng pRL-TK internal reference plasmid. Experimental groups: Each well received 200 ng of one of the following reporter vectors: pGL3-*AjCyclin E* (P1), pGL3-*AjCyclin E* (P2), pGL3-*AjCyclin E* (P3), pGL3-*AjCyclin E* (P4), or pGL3-*AjCyclin E* (P5), along with 300 ng pCMV-Flag 2C empty vector and 20 ng pRL-TK internal reference plasmid. Overexpression groups: Each well received 200 ng of one of the following reporter vectors: pGL3-*AjCyclin E* (P1), pGL3-*AjCyclin E* (P2), pGL3-*AjCyclin E* (P4), or pGL3-*AjCyclin E* (P5), together with 300 ng pCMV-Flag 2C-*AjTEAD1* and 20 ng pRL-TK internal reference plasmid. For transfection, 2 μL of Lipofectamine 6000 (Cat#C0526, Beyotime, Beijing, China) was diluted in 25 μL of DMEM medium, and the respective plasmid mixture was diluted separately in another 25 μL of DMEM medium. Both solutions were allowed to stand at 37 °C for 5 min, then combined and added to the cells. After 4 h of transfection, the medium was replaced with fresh DMEM supplemented with 10% fetal bovine serum.

### 2.10. Dual-Luciferase Reporter Assay

Dual-luciferase reporter assays were performed using the Promega kit (Cat# E1910, Promega Corporation, Madison, WI, USA) according to the manufacturer’s protocol. The procedure for measuring reporter gene promoter activity was as follows: After 48 h of transfection, first carefully aspirate the culture medium, rinse the cells twice with 200 μL of PBS, and thoroughly remove any residual PBS. Then, add 100 μL of 1× PLB lysis buffer precooled at 4 °C, and lyse the cells on ice, maintained for 30 min. After lysis, centrifuge at 4 °C and 12,000× *g* for 8 min, and collect the supernatant. Next, transfer 20 μL of cell lysate from each treatment group to an opaque 96-well plate, add 100 μL of LAR II to each well, and immediately measure the firefly luciferase fluorescence value. Afterwards, add 100 μL of Stop&Glo^®^ Reagent (Cat#E313, Promega Corporation, Madison, WI, USA) into each well and promptly recorded the Renilla luciferase signal. Finally, calculate and analyze the relative activity of the promoter luciferase.

### 2.11. Statistical Analysis

Data obtained from three independent experiments are expressed as means ± SD. Statistical differences between the control and experimental groups were determined via Student’s *t*-test. Comparisons involving three or more groups were analyzed using one-way ANOVA with Duncan’s post hoc test. The threshold for statistical significance was set at *p* < 0.05. All statistical calculations were conducted with GraphPad Prism (v7.0).

## 3. Results

### 3.1. Identification and Sequence Analysis of AjTEAD1

**Figure 1:**
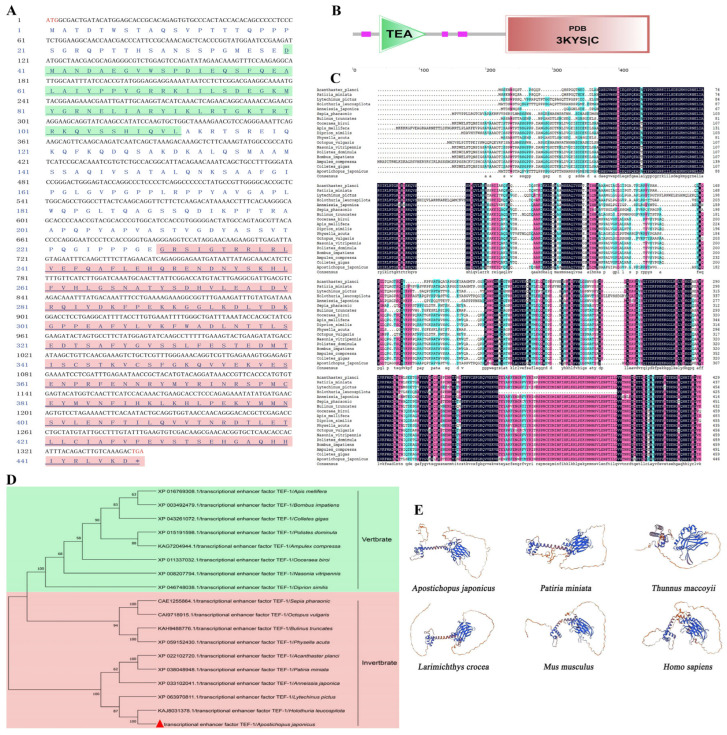
Identification and sequence analysis of AjTEAD1. (**A**) Nucleotide and cDNA-derived aa sequences of AjTEAD1. The TEA domain (40–111 aa) is shown in light green and PDB domain (229–447 aa) is shown in light red. (**B**) The domain structure of AjTEAD1 predicted by the SMART server. The TEA domain (40–111 aa) is shown in light green and PDB domain (229–447 aa) is shown in light red. (**C**) Multiple sequence alignments of AjTEAD1 across vertebrate and invertebrate species, including *Acanthaster planci*, *Patiria miniata*, *Lytechinus pictus*, *Holothuria leucospilota*, *Anneissia japonica*, *Sepia pharaonis*, *Bulinus truncatus*, *Ooceraea biroi*, *Apis mellifera*, *Diprion similis*, *Physella acuta*, *Octopus vulgaris*, *Nasonia vitripennis*, *Polistes dominula*, *Bombus impatiens*, *Ampulex compressa*, *Colletes gigas*. Columns that are completely conserved (all sequences are the same) or strongly conserved (the vast majority of sequences are amino acids of the same nature) will be colored. (**D**) Evolutionary relation analysis among TEAD1 species using the N-J method with 10,000 replications. Vertbrate is shown in light green; Invertbrate is shown in light red. *Apostichopus japonicus* marked with a red triangle. (**E**) Predicted 3D model of TEAD1 proteins, including *Apostichopus japonicus*, *Patiria miniata*, *Thunnus maccoyii*, *Larimichthys crocea*, *Mus musculus*, *Homo sapiens*.

Through transcriptomic screening of *A. japonicus*, a full-length cDNA encoding a putative TEAD1 ortholog was identified and designated as *AjTEAD1*. The *AjTEAD1* open reading frame is 1344 bp in length and encodes a 447-amino acid polypeptide. The deduced protein exhibits a molecular weight of approximately 49.95 kDa and a pI value of 8.16 (Figure 1A). Domain architecture analysis revealed a conserved TEA domain (Asp^40^–Leu^111^) and a protein-binding domain (Gly^231^–Asp^446^) (Figure 1B). Multiple sequence alignment demonstrated that AjTEAD1 shares high sequence conservation with TEAD1 orthologs from both vertebrates and invertebrates (Figure 1C). To elucidate its evolutionary relationship, a phylogenetic tree was inferred from 18 TEAD1 amino acid sequences across a range of species. The results indicated that TEAD1 proteins exhibit a continuous evolutionary trajectory from invertebrates to vertebrates, with AjTEAD1 clustering closely with TEAD1 from other echinoderms and invertebrates such as *Holothuria leucospilota* and *Acanthaster planci* (Figure 1D). The three-dimensional (3D) structure of AjTEAD1 from both vertebrate and invertebrate species was modeled using the SWISS-MODEL server. Comparative analysis revealed that the TEAD1 domain of AjTEAD1 shares a highly conserved 3D conformation with that of other species, such as *Patiria miniata*, *Larimichthys crocea*, and *Mus musculus* (Figure 1E).

### 3.2. Preparation and Specificity Validation of AjTEAD1 Antibody

**Figure 2:**
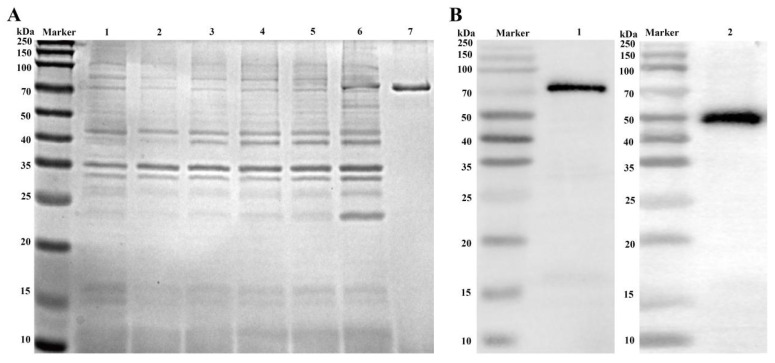
AjTEAD1 recombinant expression and antibody preparation. (**A**) The pET32a(+)-AjTEAD1 expression in *E. coli* Rosetta (DE3) was analysed by SDS-PAGE. Lane M: protein marker; Lane 1-6: IPTG induction 0, 1, 3, 5, 7 h, 12 h; Lane 7: purified AjTEAD1 protein. (**B**) Antibody specificity assessment targeting AjTEAD1. Lane 1: AjTEAD1 antibody was used to detect specificity to rAjTEAD1; Lane 2: AjTEAD1 antibody was used to detect specificity to AjTEAD1 from regenerated intestinal total protein. Original images can be found in Supplementary Materials.

To facilitate functional studies, the recombinant AjTEAD1 (rAjTEAD1) was produced in *E. coli* using the pET-32a(+) expression system and purified by Ni-NTA chromatography. SDS-PAGE analysis confirmed a distinct band near 70 kDa, corresponding to rAjTEAD1 (49.95 kDa) along with the plasmid-encoded fusion tag (Figure 2A). A polyclonal antibody against AjTEAD1 was subsequently raised in mouse. Western blot analysis confirmed the high specificity of the antibody, as it recognized both the purified rAjTEAD1 (~70 kDa) and a single endogenous protein of the expected size (49.95 kDa) in regenerating intestine of *A. japonicus* (Figure 2B).

### 3.3. AjTEAD1 Expression Is Synchronized with Cell Proliferation During Intestinal Regeneration

**Figure 3:**
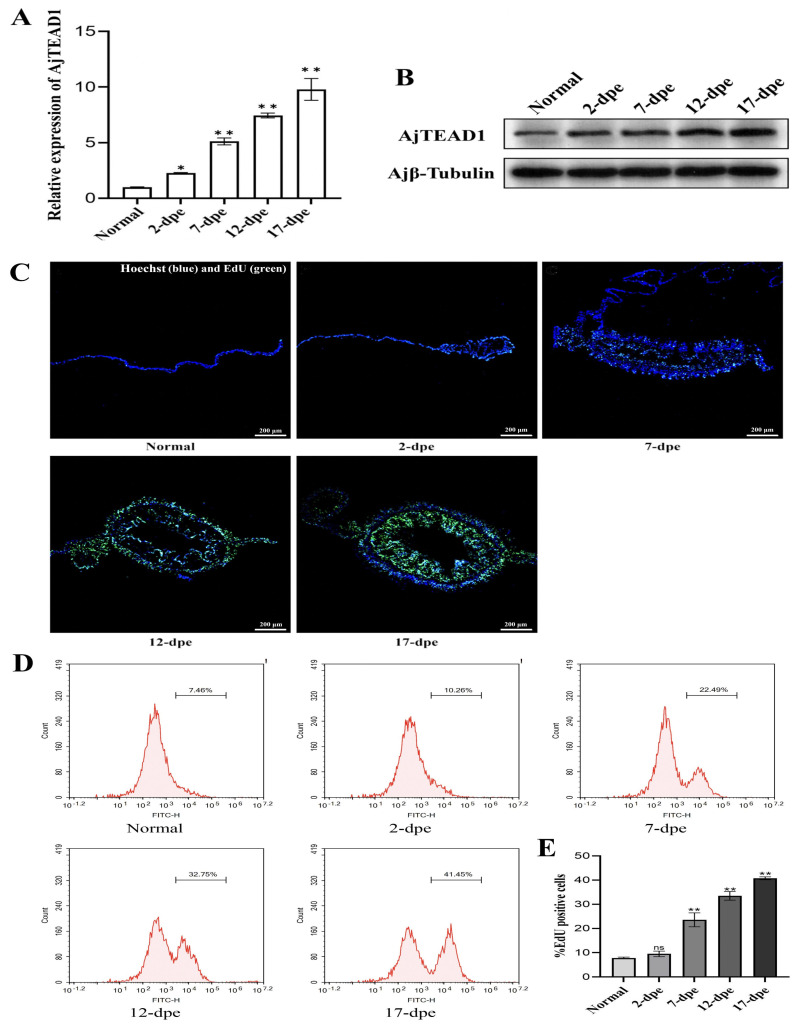
AjTEAD1 expression is synchronized with cell proliferation during intestinal regeneration. (**A**) qRT-PCR was used to validate the relative expression levels of *AjTEAD1* during the process of normal and intestinal regeneration process in *A. japonicus*. The data are presented as the mean ± SD (*n* = 3). * *p* < 0.05; ** *p* < 0.01; (**B**) Western blotting was performed to evaluate the expression changes in AjTEAD1 protein in the normal and different regenerative stage. (**C**) The cell proliferation levels were assessed through EdU- incorporation assay across both normal and regenerative phases of regeneration. Blue color indicates Hoechst-stained nuclei, and green indicates EdU-stained proliferating cells. Bar = 200 μm; (**D**) EdU-based flow cytometry was used to analysis the changes in cell proliferation levels of normal and different regenerative stages. Per sample, 10, 000 events were acquired on an flow cytometer. (**E**) Quantitative comparison of EdU-positive cells between normal and four distinct regenerative phases was conducted, with datasets visualized through column graph representations. The presented values correspond to means ± standard deviation (SD) derived from triplicate biological replicates. “ns” indicates no significant difference versus the control. ** *p* < 0.01. Original images can be found in Supplementary Materials.

To examine *AjTEAD1* expression dynamics during intestinal regeneration in *A. japonicus*, we quantified its mRNA levels in intestinal tissues at distinct regenerative phases using RT-qPCR. The result showed that compared with the control group, *AjTEAD1* mRNA was markedly increased, reaching 2.27-fold at 2-dpe, and further rising to 5.10-, 7.44-, and 9.78-fold at 7-, 12-, and 17-dpe, respectively (Figure 3A). Western blot analysis revealed that AjTEAD1 protein levels progressively increased during regeneration. Relative to controls, the fold changes reached 1.86, 2.83, 3.46, and 3.92 at 2-, 7-, 12-, and 17-dpe, respectively (Figure 3B and Figure S1). Previous studies have shown that the mesentery serves as the regeneration center of the *A. japonicus*’s intestine [5,13]. The enlargement of its free end lays the foundation for the formation of a novel intestine. This process relies on a large number of newly generated cells and involves precise regulation of cell proliferation. To determine the contribution of cell proliferation to mesenteric thickening and intestinal rudiment formation during regeneration, proliferative activity in the mesentery and rudiment was assessed via EdU immunohistochemistry and flow cytometry. The results showed that as the process of intestinal regeneration progressed, the area of regeneration primordia at the free end of the sea cucumber mesentery gradually increased, ultimately forming a new intestine (Figure 3C and Figure S2). A very small number of EDU labeled proliferating cells were observed in the normal group, and the number of proliferating cells gradually increased as the regeneration process progressed. This result is consistent with the results of flow cytometry (Figure 3D). In the normal group, the cell proliferation rate measured by flow cytometry was 7.82% ± 0.35% (Figure 3D). On 2-dpe, sporadic proliferating cells appeared, with a proliferation rate of 9.5% ± 1.13% (Figure 3D). At 7-dpe, the cell proliferation rate significantly increased to 23.56% ± 2.85%, and EdU-positive cells were mainly evenly distributed in the dilated free end of the mesentery, not limited to specific tissue layers. On 12-dpe, the proliferation rate further increased to 33.48% ± 1.79%, and the number of proliferating cells significantly increased. At 17-dpe, cavities appeared in the regenerative primordia, and the cell proliferation rate reached 40.76% ± 0.61%, indicating that the tissue was in a vigorous growth phase; at this time, the proliferating cells were mainly located in the intestinal epithelial layer, and the intestinal rudiment had basically formed. The above results indicate that the intestinal regeneration of sea cucumber is accompanied by significant cell proliferation, and the level of proliferation is not obvious at the initial stage of regeneration, but gradually increases as the regeneration progresses. Collectively, the progressive upregulation of AjTEAD1 expression closely parallels the increasing cell proliferation rate during intestinal regeneration, suggesting its potential role in regulating the proliferative processes essential for tissue regeneration.

### 3.4. Downregulation of AjTEAD1 Attenuates Cell Proliferation and Impairs Intestinal Regeneration

**Figure 4:**
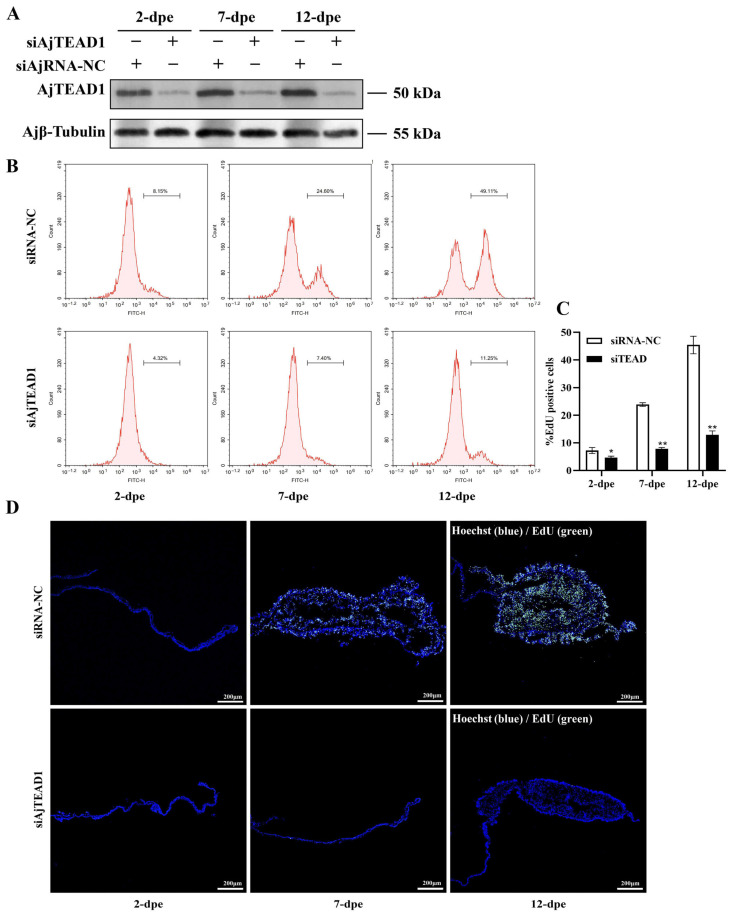
Interference with AjTEAD1 expression inhibits cell proliferation and intestinal regeneration. (**A**) Western blotting was used to detect the expression changes in AjTEAD1 protein in the regenerating mesentery and intestine at 2-, 7- and 12-days post evisceration (dpe) post siAjTEAD1 treatment. (**B**) Measurement of cell proliferation levels in the regenerative mesentery of siRNA-NC group and siAjTEAD1 treatment group at 2-, 7- and 12-dpe of A. japonicus intestinal regeneration by flow cytometry. (**C**) Quantitative comparison of EdU-positive cells between normal and four distinct regenerative phases was conducted, with datasets visualized through column graph representations. The presented values correspond to means ± standard deviation (SD) derived from triplicate biological replicates. * *p* < 0.05; ** *p* < 0.01; (**D**) EdU assay detection of cell proliferation and the size of the regenerative mesentery in the siRNA-NC group and siAjTEAD1 treatment group at 2-, 7- and 12-dpe. Blue color indicates Hoechst-stained nuclei, and green indicates EdU-stained proliferating cells. Bar = 200 μm. Original images can be found in Supplementary Materials.

To verify whether AjTEAD1 regulates cellular proliferation accompanying intestinal regeneration in *A. japonicus*, this study synthesized a specific siRNA targeting AjTEAD1 (siAjTEAD1) along with a negative control siRNA (siRNA-NC). As shown in Figure 4A and Figure S3, AjTEAD1 expression increased significantly in both the mesentery and the emerging intestinal rudiments in the siRNA-NC group, whereas it decreased markedly in the siAjTEAD1 interference group. Further detection of cell proliferation was performed using immunohistochemistry and flow cytometry to analyze EdU-labeled cells. Flow cytometry results showed significant differences in the proportion of proliferating cells between the siRNA-NC group and the siAjTEAD1 group on 2-, 7-, and 12-dpe. Specifically, the proliferation rates in the siRNA-NC group were 7.23 ± 1.1%, 23.9 ± 0.66%, and 45.42 ± 3.17%, respectively, while those in the siAjTEAD1 group were only 4.66 ± 0.53%, 7.85 ± 0.45%, and 12.92 ± 1.44% (Figure 4B,C). Immunohistochemical results (Figure 4D) were consistent with the analysis of flow cytometry. At 2-, 7- and 12-dpe, the siAjTEAD1 group exhibited a marked decrease in both the number of EdU-positive cells within the intestinal regeneration primordium and the overall size of this structure, relative to the siRNA-NC control group (Figure 4D and Figure S4). Collectively, these findings demonstrate that AjTEAD1 contributes to intestinal regeneration by modulating cellular proliferation.

### 3.5. AjTEAD1 Directly Activates Transcription of AjCyclin E

**Figure 5:**
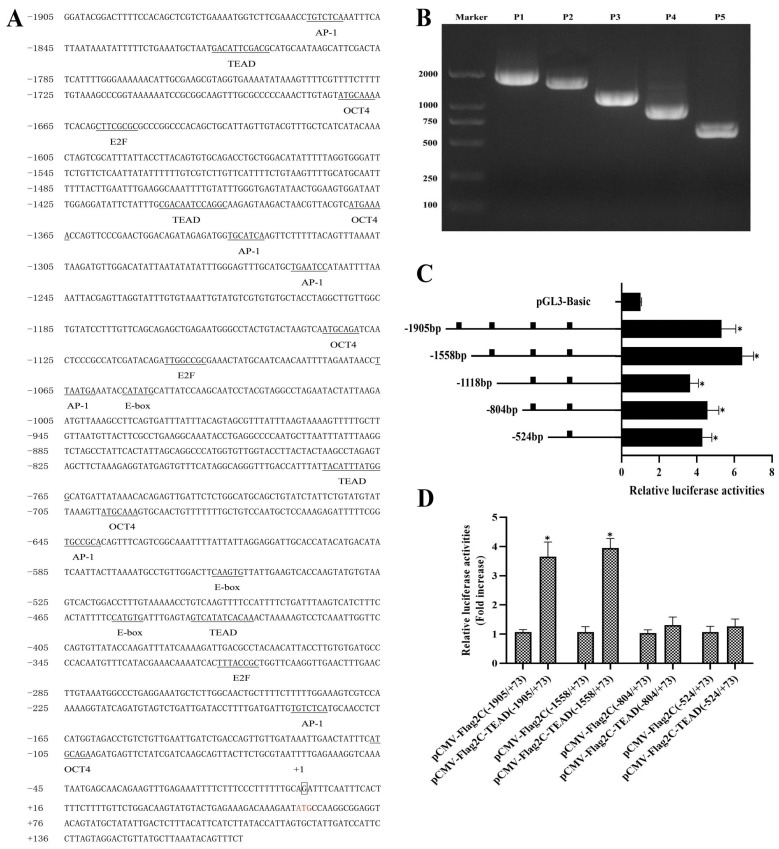
AjTEAD1 regulates the transcription of *AjCyclin E*. (**A**) Sequence information of upstream promoters for *AjCyclin E*. The transcription start site was boxed and marked as +1; The underlined sequences indicate potential transcription factor binding sites, and the TEAD1 binding sites are among these underlined regions. Red word presented the translation start site; (**B**) Electrophoresis analysis of different length of *AjCyclin E*; (**C**) Analysis of transcriptional activity after transfection of gradient-truncated *AjCyclin E* promoter fragments into HEK293T cells. The truncated *AjCyclin E* promoter regions were inserted into the pGL3-Basic reporter gene vector followed by co-transfection with pRL-TK into HEK293T cells, respectively, and the transcriptional activity of each fragment was detected by the Dual-luciferase reporter system. ■ represents potential transcription binding sites for TEAD. The data are presented as the mean ± SD (*n* = 3). * *p* < 0.05. (**D**) The effect of different regions of transcription factor TEAD1 on the transcriptional activity of *AjCyclin E* promoter was detected by Dual-luciferase reporting system. The data are presented as the mean ± SD (*n* = 3). * *p* < 0.05. Original images can be found in Supplementary Materials.

In mammals, the TEAD family transcription factors drive cell proliferation and regulate the growth of tissues and organs by directly regulating the expression of a variety of proteins closely related to cell proliferation, the cell cycle and cell survival, such as cyclins [14]. Considering the conserved function of TEAD in cell cycle regulation, we speculate that AjTEAD, a homologous gene in sea cucumber, may also participate in intestinal regeneration through a similar mechanism. In order to explore whether AjTEAD mediates the intestinal regeneration of sea cucumber by regulating the expression of cell cycle-associated proteins, we cloned the promoter regions of several cell cycle-associated genes in the genome of sea cucumber, and predicted the possible AjTEAD binding sites via the JASPAR database (https://jaspar.genereg.net, accessed on 26 March 2026). The results showed that three potential binding sites were found in the promoter region of *Ajcyclin E* (Figure 5A). Cyclin E serves as a central regulator of cell proliferation and is indispensable for normal development as well as tissue regeneration [15,16]. To elucidate the transcriptional regulation of *AjCyclin E*, we amplified a 1905 bp fragment upstream of its transcriptional start site (TSS) from sea cucumber genomic DNA. In silico analysis via the JASPAR database (https://jaspar.elixir.no, accessed on 26 March 2026) revealed multiple transcription factor binding motifs, including E2F, AP-1, and OCT4, as well as four potential TEAD binding sites at positions −1817 to −1807, −1407 to −1395, −776 to −765, and −441 to −430 (Figure 5A). To functionally characterize the *AjCyclin E* promoter, we constructed a series of truncated fragments and assessed their activities using dual-luciferase reporter assays in HEK293T cells (Figure 5B). All constructs exhibited significantly higher transcriptional activity than the empty pGL3-Basic vector, with the P2 fragment (−1508/+73) showing the strongest activity (6.41-fold increase), indicating that critical cis-regulatory elements reside within the P1 and P2 regions (Figure 5C). To determine whether AjTEAD1 directly activates *AjCyclin E* transcription, each promoter fragment was co-transfected with an AjTEAD1 overexpression plasmid. Dual-luciferase assays revealed that AjTEAD1 significantly enhanced the activity of the P1 (−1905/+73) and P2 (−1508/+73) promoters by 3.64-fold and 3.95-fold, respectively, while no significant induction was observed for the P4 (−804/+73) or P5 (−524/+73) fragments (Figure 5D). These findings indicate that the TEAD binding site located between −1407 and −1395 is essential for AjTEAD1-mediated transactivation of *AjCyclin E*.

### 3.6. AjCyclin E Is a Key Effector of Cell Proliferation Downstream of AjTEAD1

**Figure 6:**
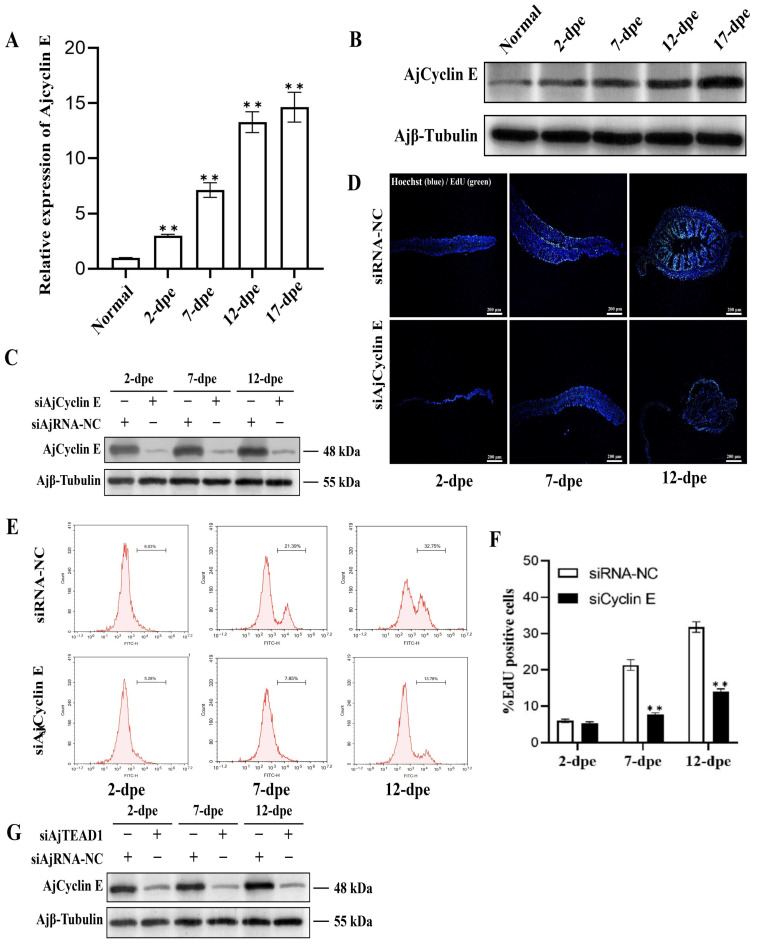
AjCyclin E acts downstream of AjTEAD1 to facilitate intestinal regeneration. (**A**) qRT-PCR was used to validate the relative expression levels of *AjCyclin E* during the process of normal and intestinal regeneration in *A. japonicus*. The data are presented as the mean ± SD (*n* = 3). ** *p* < 0.01; (**B**) Western blotting was performed to evaluate the expression changes in AjCyclin E protein in the normal and different regenerative stage. (**C**) Western blotting was used to detect the expression changes in AjCyclin E protein in the regenerating mesentery and intestine at 2-, 7- and 12-dpe post siCyclin E treatment; (**D**) EdU assay detection of cell proliferation and the size of the regenerative mesentery in the siRNA-NC group and siCyclin E treatment group at 2-, 7- and 12-dpe. Blue color indicates Hoechst-stained nuclei, and green indicates EdU-stained proliferating cells. Bar = 200 μm; (**E**) Measurement of cell proliferation levels in the regenerative mesentery of siRNA-NC group and siCyclin E treatment group at 2-, 7- and 12-dpe of *A. japonicus* intestinal regeneration by flow cytometry. Per ample, 10, 000 events were acquired on an flow cytometer. (**F**) Quantitative comparison of EdU-positive cells was conducted, with datasets visualized through column graph representations. The presented values correspond to means ± standard deviation (SD) derived from triplicate biological replicates. ** *p* < 0.01. (**G**) Western blotting was used to detect the expression changes in AjTEAD1 protein in the regenerating mesentery and intestine at 2-, 7- and 12-dpe post siAjTEAD1 treatment. Original images can be found in Supplementary Materials.

We next examined the expression and function of AjCyclin E during regeneration. Similar to AjTEAD1, both AjCyclin E transcript and protein abundances were significantly upregulated in a time-dependent manner during intestinal regeneration (Figure 6A,B and Figure S5). To determine its functional role, we knocked down AjCyclin E in vivo using a specific siRNA (siAjCyclin E), which resulted in a significant reduction in its protein expression (Figure 6C and Figure S6). Mirroring the AjTEAD1 knockdown phenotype, inhibition of AjCyclin E led to a significant decrease in the size of the regenerating intestinal primordium and a marked reduction in the number of EdU-positive proliferating cells at 7 dpe and 12 dpe (Figure 6D and Figure S7). Flow cytometry analysis confirmed this, showing that the proliferation rate in the siAjCyclin E group was significantly lower than in the siRNA-NC group at 7 dpe (7.65% ± 0.55% vs. 21.32% ± 1.44%) and 12 dpe (14.06% ± 0.69% vs. 31.77% ± 1.43%) (Figure 6E,F). More importantly, we detected that knockdown of AjTEAD1 resulted in a concomitant downregulation of AjCyclin E protein expression (Figure 6G and Figure S8), placing Cyclin E genetically downstream of TEAD1. Collectively, these data demonstrate that AjTEAD1 promotes cell proliferation during intestinal regeneration by directly activating the transcription of its target gene, *AjCyclin E*.

## 4. Discussion

Sea cucumbers (Holothuroidea) are commercially and ecologically valuable marine echinoderms, best known for their dramatic capacity for visceral autotomy (evisceration) and complete intestinal regeneration. Evisceration is triggered by a series of environmental and biological stressors. In *A*. *japonicus*, it occurs through cloacal rupture to expel the digestive tract, respiratory trees and gonads. This behavior is usually adaptive, such as defending against predation or eliminating parasites [17]. However, pathogen-induced evisceration in diseased animals is a pathological cascade related to bacterial, parasitic or viral challenges, and it causes high mortality in aquaculture. In this context, understanding the regenerative response is critical. Nowadays, Research on sea cucumber intestinal regeneration has advanced considerably. Studies have revealed the histological processes of mesenterial thickening and intestinal lumen recanalization, the dynamic changes in multiple cellular events including cell migration, autophagy, dedifferentiation, and proliferation, as well as conserved signaling pathways that regulate tissue regeneration [5,12]. Despite these advances, the molecular regulators governing the tightly controlled proliferative burst that underpins regeneration remain incompletely understood. Elucidating these mechanisms is not only essential to unlocking the extraordinary regenerative capacity of echinoderms but also offers potential insights into improving resilience in commercial sea cucumber species challenged by disease-induced evisceration.

Cell proliferation is the driving force behind tissue regeneration, generating new cells to replace lost or damaged structures [18]. Consistent with established literature, a tightly regulated burst of proliferative activity represents a critical initial phase in most regenerative paradigms, without which subsequent differentiation, matrix deposition, and remodeling cannot proceed effectively, often resulting in compromised repair or scar formation [19,20]. In the present study, we observed a progressive upregulation of proliferation during intestinal regeneration in *A. japonicus*, with minimal activity at early stages and a peak reached at 17 dpe. EdU staining assays further revealed a clear spatial and temporal correlation between proliferation and the expansion of the free end of the mesentery. Specifically, low proliferation in the initial phase corresponded with no noticeable mesenteric thickening, whereas robust proliferation in mid-to-late stages coincided with pronounced enlargement of the mesenteric terminus. Given that mesenteric expansion underlies the formation of the intestinal regeneration primordium, we propose that newly generated cells from this proliferative burst constitute the primary cellular source for mesenteric enlargement, thereby playing a significant role in establishing the intestinal regenerative primordium.

Cell proliferation is regulated by conserved signaling pathways such as *Wnt*/*β-catenin*, *Hedgehog*, *Notch*, *Hippo*, and growth factors such as FGF and EGFR [21,22,23]. Among them, the TEAD transcription factor family, as a key downstream effector component of the Hippo pathway, has a role in regulating cell proliferation in the context of tissue regeneration [24]. To investigate the regulatory mechanism of AjTEAD1 on *A*. *japonicus* cell proliferation and intestinal regeneration, this study successfully cloned the ORF of *AjTEAD1*. Sequence analysis shows that *AjTEAD1* encodes 447 amino acids, and the SMART domain predicts that it contains an N-terminal TEA domain (Asp^40^–Leu^111^) and a C-terminal protein binding domain (Gly^231^–Asp^446^). The TEA domain is a signature DNA binding region of the TEAD protein family, responsible for recognizing MCAT elements on target gene promoters and transmitting transcriptional activation signals through interactions with co transcription factors such as YAP/TAZ [25]; The C-terminal domain is responsible for recruiting other transcription regulatory factors, which is consistent with the structural characteristics of TEAD proteins reported in previous studies. TEAD family members are widely recognized as key regulators of tissue growth and regeneration across various animal groups [24], with conserved functions in driving regenerative cell proliferation. For example, in the mouse myocardial regeneration model, activation of the TEAD1-YAP complex can significantly promote the proliferation of adult mammalian cardiomyocytes, while TEAD1 deficiency can lead to cardiac developmental defects [26]; in the repair of mouse neurological injuries, TEAD1 participates in the injury response by regulating the differentiation of glial cells [27]; in addition, recent studies have found that TEAD1 can accelerate skin wound healing and spinal cord injury repair by promoting the secretion of stem cell exosomes [28]. In terms of intestinal regeneration, studies have shown that the YAP-TEAD complex is crucial for the maintenance and proliferation of adult stem cells during amphibian intestinal remodeling and mammalian intestinal regeneration processes [29]. These findings indicate that TEAD proteins play conserved roles in regulating tissue regeneration across different species, and our study further reveals the important role of AjTEAD1 in sea cucumber intestinal regeneration, providing new insights into the functional conservation of TEAD family proteins in regenerative processes.

Cyclin E is a core component of the cell cycle regulatory network that governs the G1/S transition, a critical checkpoint for initiating DNA replication and cell proliferation [30]. Its pivotal role in development and regeneration has been well-documented across metazoans, with its expression tightly linked to tissue regeneration and repair processes [31]. In our study, the observed temporal upregulation of AjCyclin E during intestinal regeneration in sea cucumbers, correlating with increased cell proliferation, prompted us to investigate its upstream regulatory mechanism. In silico analysis of the *AjCyclin E* promoter region revealed the presence of four putative TEAD-binding sites among other transcription factor motifs, suggesting a potential direct link to the *Hippo* signaling pathway. Subsequent dual-luciferase reporter assays confirmed that the proximal promoter region (−1905 to +73 bp) possesses strong transcriptional activity, and this activity was significantly enhanced upon AjTEAD1 overexpression. Importantly, this transactivation was dependent on the integrity of the TEAD-binding sites, as deletion constructs lacking these elements (P4 and P5) failed to respond to AjTEAD1. This provides compelling evidence that AjTEAD1 directly regulates *AjCyclin E* transcription. Our functional data further support this model, as knockdown of AjTEAD1 led to a significant reduction in both AjCyclin E transcript levels and cellular proliferation, reinforcing that AjTEAD1 is a necessary upstream regulator of this cell cycle engine during regeneration.

## 5. Conclusions

Based on comprehensive functional and molecular characterization, AjTEAD1 is a pivotal regulator of intestinal regeneration in the sea cucumber *A*. *japonicus*. Its conserved structural domains underpin its core role in driving regenerative tissue repair by promoting cell proliferation, via a direct molecular mechanism: injury-induced upregulation of AjTEAD1 enables its specific binding to the *AjCyclin E* promoter, activating transcriptional expression of this key cell cycle gene. Interfering with AjTEAD1 expression reduces AjCyclin E levels, which suppresses cell proliferation and ultimately compromises intestinal regeneration. These findings identify the *TEAD–Cyclin E* axis as a conserved core regulatory module in echinoderm regeneration, filling a critical knowledge gap in aquatic invertebrate regenerative biology. Beyond advancing fundamental understanding of metazoan regenerative mechanisms, this work elucidates the regenerative cytology of an economically important echinoderm, and highlights AjTEAD1 as a key target for exploring and modulating regenerative capacity in sea cucumbers and related aquatic invertebrates.

## Figures and Tables

**Table 1:** Primers used in this study.

Primer Name	Primer Sequence (5′-3′)	Used for
AjTEAD1-F AjTEAD1-R	GCTGATATCGGATCCGAATTCATGGCGACTGATACATGGAGC TGGTGGTGGTGGTGCTCGAGTGTCTTTGACAAGTCTGTAAATG	Recombinant expression
AjTEAD1-pCMV-F AjTEAD1-pCMV-R	AAGGACGACGATGACAAGCTTATGGCGACTGATACATGGAGC ATGTCTGGATCCCCGCGGCCGCTCAGTCTTTGACAAGTCTGTA	
AjCyclin E-F AjCyclin E-R	ATCCTTTCTGTCCAGTTACC ACCTTTCGTGCGTGTTTC	Promoter check
P1 (−1905/+73)	TTTCTCTATCGATAGGTACCGGATACGGACTTTTCCACAG	Promoter activity
P2 (−1558/+73)	TTTCTCTATCGATAGGTACCTTTAGGTGGGATTTCTGTTC	
P3 (−1118/+73)	TTTCTCTATCGATAGGTACCCATCGATACAGATTGGCC	
P4 (−804/+73)	TTTCTCTATCGATAGGTACCGTTTCATAGGCAGGGTTTGAC	
P5 (−524/+73)	TTTCTCTATCGATAGGTACCTCACTGGACCTTTGTAAAAACCT	
P (+73)	TTACTTAAGATCGCAGATCTCGAGCTCCGCCTTGGCATATTCT	
qAjTEAD1-F qAjTEAD1-R	TGTGGGAGGAGGAAAATAAT TGCCTGTTCTGAGTTTGATG	Real-time qPCR
qAjCyclin E-F qAjCyclin E-R	CACAACATCCAAACACATTC GCCTTCCTCTTCTTCTTACTAC	
qAjβ-tubulin-F qAjβ-tubulin-R	GCACATCAAGCCGTCAAACTCAC TATGCCCGCATAGCAAACATACC	
siAjTEAD1	GCACCUCCCAGAGAAAUAUTT AUAUUUCUCUGGGAGGUGCTT	RNA interference
siAjCyclin E	GCUCACUUAUGAAGACAUUTT AAUGUCUUCAUAAGUGAGCTT	
siRNA (NC)	UUCUCCGAACGUGUCACGUTT ACGUGACACGUUCGGAGAATT	

The underline letters represent homologous arms or restriction enzyme sites.

**Table 2:** Antibodies used in this study.

Antibodies	Isotype	Use	Product No.	Source
Anti-Cyclin E antibody	Rabbit	WB (1:1000)	11554-1-AP	Proteintech
Anti-β-tubulin monoclonal antibody	Mouse	WB (1:5000)	M20005S	Abmart
HRP-conjugated goat anti-mouse IgG	Mouse	WB (1:5000)	D110087	BBI
HRP-conjugated goat Anti-rabbit IgG	Rabbit	WB (1:5000)	D110058	BBI

## Data Availability

The original contributions presented in this study are included in the article. Further inquiries can be directed to the corresponding authors.

## Supplementary Materials

The following supporting information can be downloaded at: https://www.mdpi.com/article/10.3390/biom16050642/s1. Figure S1: Band density of AjTEAD1 proteins in Figure 3B was quantified using the ImageJ program; Figure S2: Regeneration primordia in Figure 3C were quantified using ImageJ. Figure S3: Band density of AjTEAD1 proteins in Figure 4A was quantified using the ImageJ program. Figure S4: Regeneration primordia in Figure 4D were quantified using ImageJ. Figure S5: Band density of AjCyclin E proteins in Figure 6B was quantified using the ImageJ program. Figure S6: Band density of AjCyclin E proteins in Figure 6C was quantified using the ImageJ program. Figure S7: Regeneration primordia in Figure 6D were quantified using ImageJ. Figure S8: Band density of AjCyclin E proteins in Figure 6G was quantified using the ImageJ program. Original images of Figure 3B, Figure 4A and Figure 6B,C,G are provided in the Supplementary Materials.
