# Supplementary material for: AjTEAD1 Targets AjCyclin E to Promote Cell Proliferation During Intestinal Regeneration in Apostichopus japonicus
Source: Biomolecules. 2026 Apr 25;16(5):642. doi: 10.3390/biom16050642 (PMC13204635; doi:10.3390/biom16050642)
Supplement: Supplementary file 1 [file biomolecules-16-00642-s001.zip › biomolecules-4254501-supplementary.pdf]

Figure S1

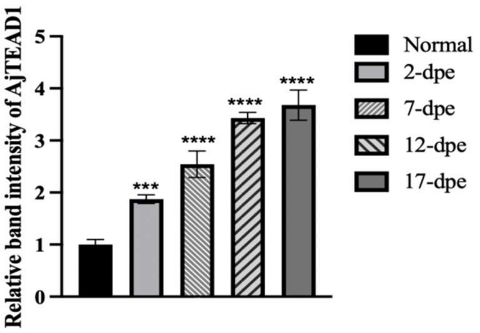

Figure S1. Band density of AjTEAD1 proteins in Figure 3B was quantified using the ImageJ program. Data are presented as mean ± SD, n = 3 replicates. Statistical significance was assessed using Student's t-test: ***$p < 0.001$, ****$p < 0.0001$.

Figure S2

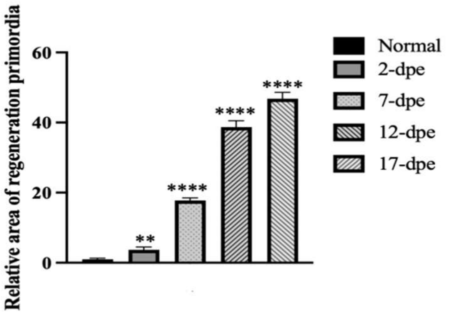

Figure S2. Regeneration primordia in Figure 3C were quantified using ImageJ. Data are presented as mean ± SD, n = 3 replicates. Statistical significance was assessed using Student's t-test: **$p < 0.01$, ****$p < 0.0001$.

Figure S3

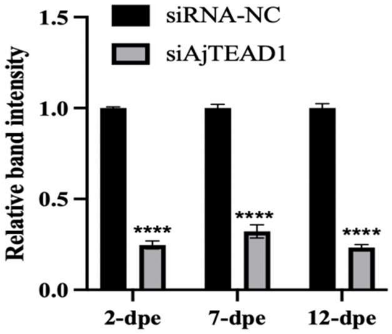

Figure S3. Band density of AjTEAD1 proteins in Figure 4A was quantified using the ImageJ program. Data are presented as mean ± SD, n = 3 replicates. Statistical significance was assessed using Student's t-test: ****$p < 0.0001$.

Figure S4

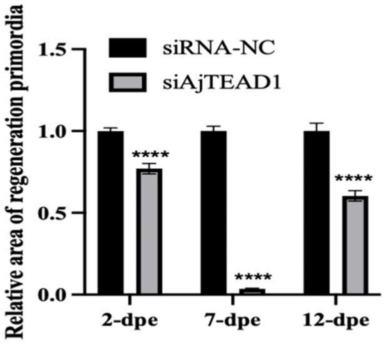

Figure S4. Regeneration primordia in Figure 4D were quantified using ImageJ. Data are presented as mean ± SD, n = 3 replicates. Statistical significance was assessed using Student's t-test: ****$p < 0.0001$.

Figure S5

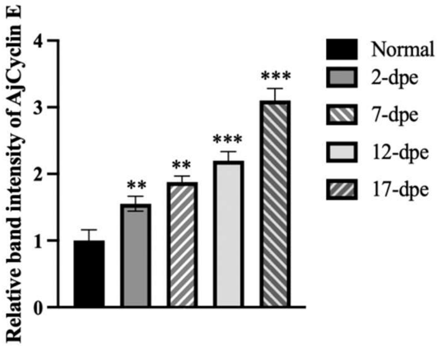

Figure S5. Band density of AjCyclin E proteins in Figure 6B was quantified using the ImageJ program. Data are presented as mean ± SD, n = 3 replicates. Statistical significance was assessed using Student's t-test: **$p < 0.01$, ***$p < 0.001$.

Figure S6

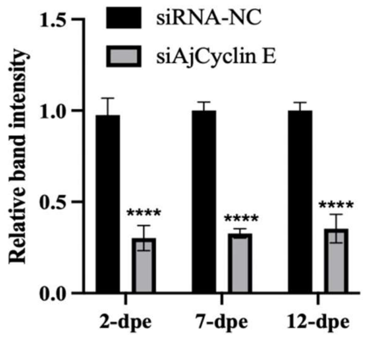

Figure S6. Band density of AjCyclin E proteins in Figure 6C was quantified using the ImageJ program. Data are presented as mean ± SD, n = 3 replicates. Statistical significance was assessed using Student's t-test: ****$p < 0.0001$.

Figure S7

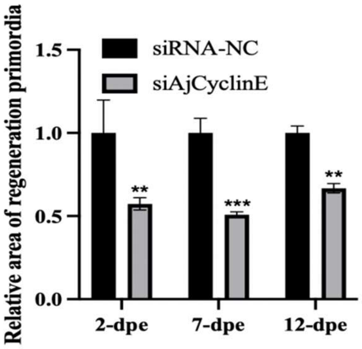

Figure S7. Regeneration primordia in Figure 6D were quantified using ImageJ. Data are presented as mean ± SD, n = 3 replicates. Statistical significance was assessed using Student's t-test: **$p < 0.01$, ***$p < 0.001$.

Figure S8

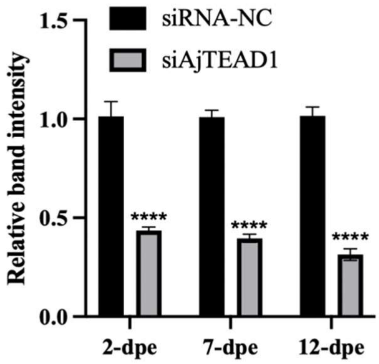

Figure S8. Band density of AjCyclin E proteins in Figure 6G was quantified using the ImageJ program. Data are presented as mean ± SD, n = 3 replicates. Statistical significance was assessed using Student's t-test: ****$p < 0.0001$.

WB original images

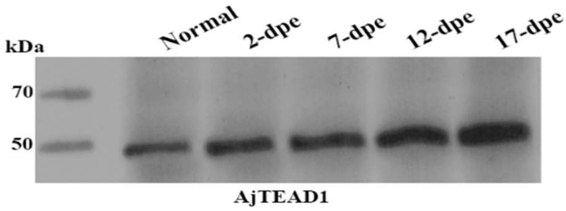

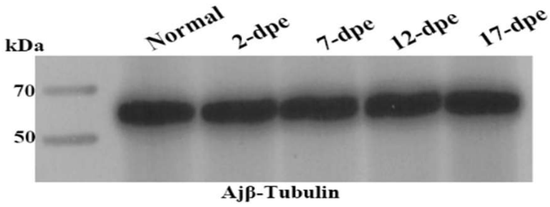

Original images of Figure 3B. Western blotting was performed to evaluate the expression changes of AjTEAD1 protein in the normal and different regenerative stage.

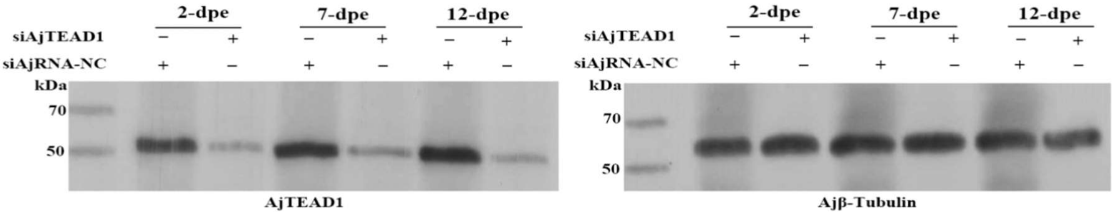

Original images of Figure 4A. Western blotting was used to detect the expression changes of AjTEAD1 protein in the regenerating mesentery and intestine at 2-, 7- and 12-dpe post siAjTEAD1 treatment.

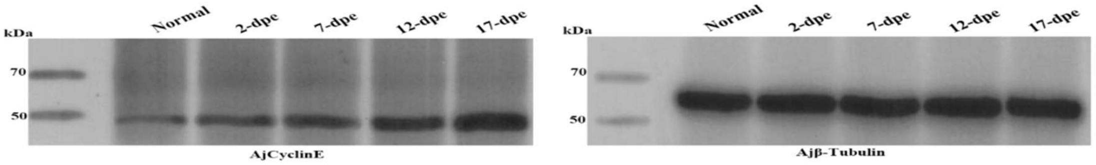

Original images of Figure 6B. Western blotting was performed to evaluate the expression changes of AjCyclin E protein in the normal and different regenerative stage.

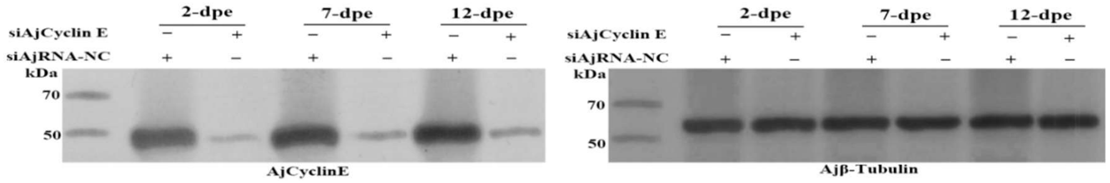

Original images of Figure 6C. Western blotting was used to detect the expression changes of AjCyclin E protein in the regenerating mesentery and intestine at 2-, 7- and 12-dpe post siCyclin E treatment

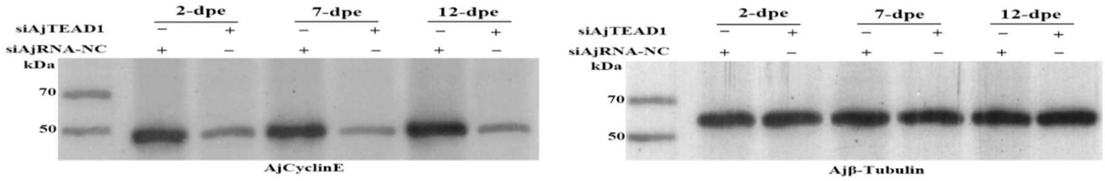

Original images of Figure 6G. Western blotting was used to detect the expression changes of AjTEAD1 protein in the regenerating mesentery and intestine at 2-, 7- and 12-dpe post siAjTEAD1 treatment.
